# Bilateral variation in the branching pattern of the subclavian artery: an unusual finding with clinical implications

**DOI:** 10.1590/1677-5449.202201472

**Published:** 2023-03-27

**Authors:** Naveen Kumar, Vijay Samuel Paul, Kumar M. R. Bhat, Ashwini P. Aithal

**Affiliations:** 1 RAK Medical & Health Sciences University, Ras Al Khaimah College of Medical Sciences, Ras Al Khaimah, United Arab Emirates.; 2 Manipal Academy of Higher Education, Department of Basic Medical Sciences, Manipal, India.

**Keywords:** subclavian artery, thyrocervical trunk, variation, lateral cervical, cervicodorsoscapular trunk, artéria subclávia, tronco tireocervical, variação, cervical lateral, tronco escapular cervical dorsal

## Abstract

The subclavian artery is a significant branch of the aortic arch. We present a rare case of a bilateral variation in the branching pattern of the subclavian artery, observed in an adult male cadaver aged 70 years. On both the sides of the neck, all the branches of the subclavian artery took their origin from its first part. There was a rare occurrence of a cervicodorsoscapular trunk, which gave rise to superficial cervical, suprascapular, and dorsal scapular arteries. The same branching pattern was observed on the left side of the neck, with the presence of another cervicodorsoscapular trunk. Thyrocervical trunk and transverse cervical artery were both absent from the cervical region bilaterally. The inferior thyroid artery was a direct branch from the subclavian artery. Knowledge regarding variations of the subclavian artery is very important as lateral cervical region arteries are important for flap harvesting in plastic and reconstruction surgery. Preoperative radiologic evaluation of pedicles might help in choosing the optimal flap design, prevent ischemic complications, and help to improve overall treatment outcomes.

## Resumo

A artéria subclávia é um ramo significativo do arco da aorta. Apresentamos um caso raro de variação bilateral do padrão de ramificação da artéria subclávia, observada em um cadáver adulto do sexo masculino de 70 anos. Em ambos os lados do pescoço, todos os ramos da artéria subclávia originavam-se de sua primeira parte. Houve rara ocorrência de tronco escapular cervical dorsal, que deu origem às artérias cervical superficial, supraescapular e escapular dorsal. O mesmo padrão de ramificação foi observado no lado esquerdo do pescoço, com a presença de tronco escapular cervical dorsal. O tronco tireocervical e a artéria cervical transversa estavam ausentes em ambas as regiões cervicais direita e esquerda. A artéria tireóidea inferior consistia em um ramo direto da artéria subclávia. O conhecimento das variações da artéria subclávia é fundamental, pois as artérias da região cervical lateral são importantes para a obtenção de retalhos em cirurgias plásticas e reconstrutivas. A avaliação radiológica pré-operatória dos pedículos pode ajudar na escolha do desenho ideal do retalho, prevenir complicações isquêmicas e ajudar a melhorar o resultado geral do tratamento.

## INTRODUCTION

The lateral cervical region or posterior triangle of neck is located between the sternocleidomastoid muscle anteriorly and trapezius muscle posteriorly. This region is widely used for harvesting flaps and for vascularized supraclavicular lymph node transfer and is involved in angiographic investigations and radical and modified neck surgeries.^1^^,^^2^ A literature review reveals frequent changes in the anatomical nomenclature of the arteries in the lateral cervical region. Such changes, along with inadequate knowledge regarding the gross anatomy and variations in the branching pattern of the arteries, have culminated in difficulties in plastic and reconstructive surgery, especially in relation to musculocutaneous flap planning.^3^

The subclavian artery is the principal artery of the upper limb and it also gives off branches that supply the neck region. The right subclavian artery arises from the brachiocephalic trunk and the left subclavian is a branch from the arch of the aorta. The branches of the subclavian artery are the vertebral artery, internal thoracic artery, thyrocervical trunk, costocervical artery, and dorsal scapular artery. On the left side of the neck, all branches except the dorsal scapular arise from its first part, on the right side, the costocervical artery usually arises from the second part.^4^

The suprascapular artery (SSA), superficial cervical artery (SpCA), and dorsal scapular artery (DSA) are the main arteries frequently used for flap harvesting. These arteries may take their origin from the subclavian artery (SCA) and thyrocervical trunk individually or by forming common trunks and therefore have varying origin patterns. Hence, it is important to evaluate and record any arterial variations to reduce the risk of injuring these vessels during surgical interventions. In view of its immense clinical implications, an unusual bilateral variation in the branching pattern of the subclavian artery is reported and the related literature is reviewed.

## CASE REPORT

During routine dissection, a bilateral variant branching pattern of the subclavian artery was observed in an adult male cadaver aged approx.70 years. No medical history was available on these cadavers. This study complies with the Helsinki Declaration and with local ethical guidelines. The authors certify that they have obtained all appropriate consent forms and ethics committee clearance for the use of cadavers in this study. No patient data were used in the study.

**Figure 1:**
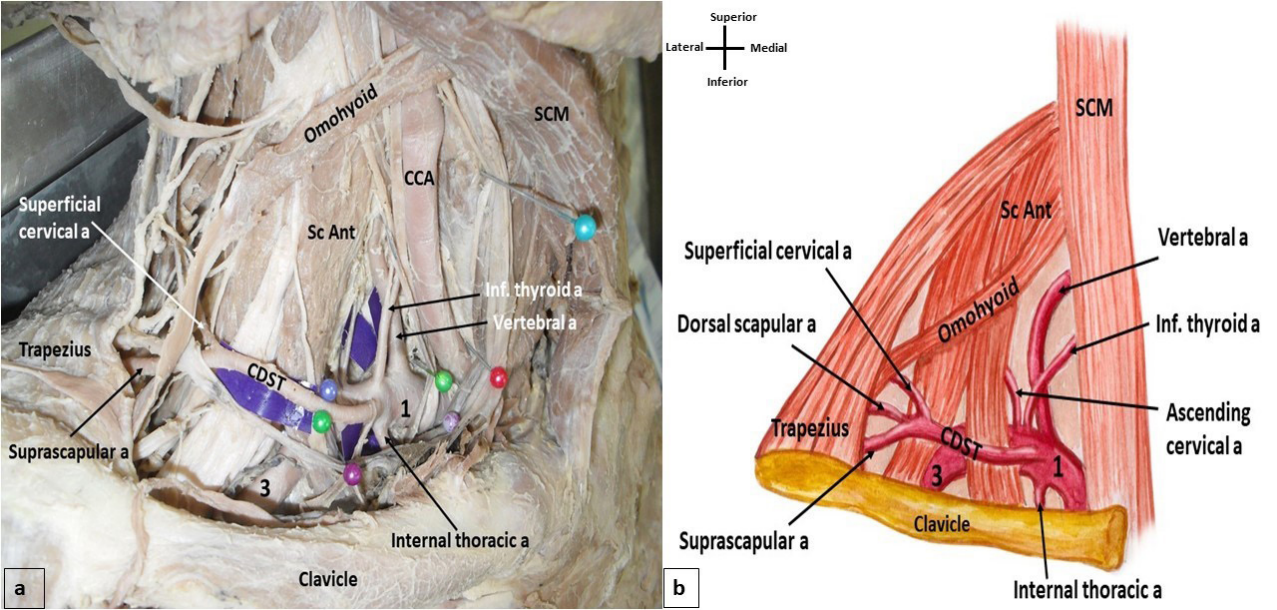
(a) Dissection of the right cervical region showing the variant branching pattern of the subclavian artery. CDST-cervicodorsoscapular trunk, SCM- sternocleidomastoid muscle, CCA-common carotid artery, Sc Ant- scalenus anterior, 1-first part of subclavian artery, 3-third part of subclavian artery; (b) Schematic diagram showing the variant branching pattern.

**Figure 2:**
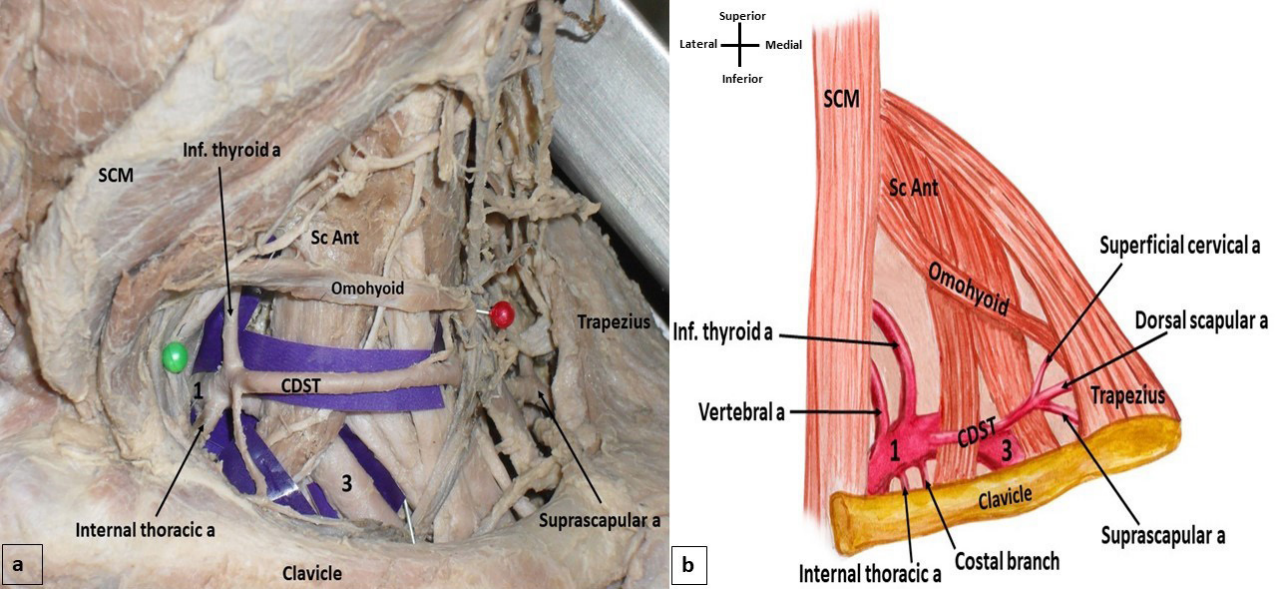
(a) Dissection of the left cervical region showing the variant branching pattern of the subclavian artery. CDST-cervicodorsoscapular trunk, SCM- sternocleidomastoid muscle, Sc Ant- scalenus anterior, 1-first part of subclavian artery, 3-third part of subclavian artery; (b) Schematic diagram showing the variant branching pattern.

The right subclavian artery took its origin from the brachiocephalic trunk, and the left subclavian artery took its origin from the aortic arch. After their origins, the subclavian arteries appeared in the lateral cervical region behind the scalenus anterior muscle and continued into the cervico-axillary canal. The scalenus anterior muscle divided the subclavian artery into three parts and variations were noted in the branches of the artery. On the right side of the neck, both thyrocervical trunk and transverse cervical artery were absent and all the branches of the subclavian artery took their origin from its first part. The inferior thyroid artery was a direct branch from the subclavian artery. There was a rare occurrence of a cervicodorsoscapular trunk (CDST) (Figure 1). The same branching pattern was also observed in the left lateral cervical region. The left thyrocervical trunk and the left transverse cervical artery were both absent, the inferior thyroid artery was a direct branch from the subclavian artery, with presence of a CDST. All branches of the subclavian artery took their origin from its first part (Figure 2). On both sides, the anomalous CDST took its origin from the first part of the subclavian artery, moved posteriorly parallel to the clavicle, and coursed anterior to the scalenus anterior muscle. At the posterior border of the scalenus medius muscle, this common trunk gave rise to superficial cervical, suprascapular, and dorsal scapular arteries, just anterior to the trapezius muscle (Figures 1 and 2).

## DISCUSSION

Variations have been reported in the origins and branching patterns of the subclavian artery and its branches. Although deviation from the normal branching pattern is not unusual, bilateral CDST with absence of thyrocervical trunk, as found in the present case, is infrequent. It is important to bear these anatomical variations in mind due to the high frequency with which the lateral cervical region is involved in diagnostic and surgical procedures.^5^ A previous study reported absence of the thyrocervical trunk in one specimen, citing it as a very rare occurrence.^6^ In their study of the subclavian artery, Bulbul et al. grouped branching patterns into 5 types, four of which involved formation of common arterial trunks. They reported presence of a CDST arising from a thyrocervical trunk in 12.5% of the cases they studied.^7^ There are limited reports of a CSDT with origin from the subclavian artery. A CSDT arising directly from the subclavian artery had a very low incidence of 1.1%^7^ and 3%.^3^ This indicates that this occurrence of bilateral CDST is a unique finding with associated clinical implications. Familiarity with the anatomy of vascular variations is therefore necessary for clinicians involved in flap reconstruction and other surgical interventions.

Embryologically, all main vessels develop from a primary plexus of smaller ones. During development, some vessels enlarge and form definitive channels and others regress. During this phase of development, it is possible that different patterns in the vessels may appear, including both the origin and/or the course of either arteries or veins.^8^

Flaps including arteries of the lateral cervical region are used in different procedures such as vascularized lymph node transfer and head and neck reconstructions.^9^ The musculocutaneous flaps, such as the flap of the lower trapezius^10^ and the dorsal scapular flap,^11^ are dependent on the integrity and functional availability of the dorsal scapular artery. Likewise, the cervico-dorsal and cervico-scapular flaps^12^ are based on the superficial cervical artery. We assume that the mode of origin of these arteries, especially their origin directly from the subclavian artery through a common trunk, will have consequences for their vascular dynamics. These variations and their limited availability can result in good or bad viability of the irrigated flaps. Preoperative radiologic evaluation of pedicles may improve the safety and success of flap reconstruction and prevent ischemic or circulatory complications during vascular surgeries involving the arteries of the lateral cervical region.

Clavicle fractures tend to be extremely common and break near the midpoint of the bone.^2^ Anomalous presence and course of the CDST, as found in the present case, could increase the danger of its being damaged by a broken clavicle. Since the suprascapular artery originates from the CDST, the muscles of the rotator cuff group could be deprived of blood flow if the CDST becomes congested or kinked. In such a case, most of the blood supply to the shoulder region could be compromised. It should be noted that anatomical variations of the suprascapular artery must be considered while performing suprascapular artery ligation for neck lymphoma containment.^13^ Knowledge regarding such rare occurrence of CDST is very helpful in avoiding damage to the arterial trunk during lymph node biopsy or caring for trauma in the posterior triangle of the neck.

## CONCLUSION

Coexistence of absence of thyrocervical trunk and anomalous presence of bilateral cervicodorsoscapular trunk is an infrequent finding. The anatomical variations presented are clinically important for making accurate diagnoses, treating clavicle fractures, and safely performing surgical procedures such as flap reconstructions. Careful examination and knowledge of anatomic variants are essential for interventional procedures involving the thorax, neck, and head.

## References

[1] Kim SY, Lee KT, Mun GH (2016). Reliable harvest of a dorsal scapular artery perforator flap by augmenting its perfusion. Microsurgery.

[2] Panagouli E, Venieratos D, Pyrgakis P (2013). Anomalous origin and course of the suprascapular artery combined with absence of the suprascapular vein: case study and clinical implications. N Am J Med Sci.

[3] Weiglein AH, Moriggl B, Schalk C, Künzel KH, Müller U (2005). Arteries in the posterior cervical triangle in man. Clin Anat.

[4] Standring S (2008). Gray’s Anatomy: the anatomical basis of clinical practice..

[5] Vorster W, du Plooy PT, Meiring JH (1998). Abnormal origin of internal thoracic and vertebral arteries. Clin Anat.

[6] Yücel AH, Kizilkanat E, Özdemir CÖ (1999). The variations of the subclavian artery and its branches. Okajimas Folia Anat Jpn.

[7] Bulbul E, Yanik B, Akay E, Koksal V, Demirpolat G (2019). Arterial variations within the lateral cervical region: a multidetector CT angiography study. Int J Morphol.

[8] Barry A (1951). The aortic arch derivatives in human adult. Anat Rec.

[9] Atallah S, Guth A, Chabolle F, Bach CA (2015). Supraclavicular artery island flap in head and neck reconstruction. Eur Ann Otorhinolaryngol Head Neck Dis.

[10] Weiglein AH, Haas F, Pierer G (1996). Anatomic basis of the lower trapezius musculocutaneous flap. Surg Radiol Anat.

[11] Ikka L, Mihalea C, Achour NB, Khalek HA, Vacher C (2016). The origin of the dorsal scapular artery: anatomic variations and surgical applications. Surg Radiol Anat.

[12] Hyakusoku H, Yoshida H, Okubo M, Hirai T, Fumiiri M (1990). Superficial cervical artery skin flaps. Plast Reconstr Surg.

[13] Memar SA, Allison JM, Frolov A, Tan Y (2019). Rare bilateral origin variations of the suprascapular arteries. Int J Anat Var.

